# Prognostic evaluation from different types of acidosis in high-risk surgical patients

**DOI:** 10.1186/cc14718

**Published:** 2015-09-28

**Authors:** João Manoel Silva Junior, Amanda Maria RR de Oliveira, Cristina P Amendola, Fernando A Mendes, LM Sá Malbouisson, Maria José C Carmona, Pedro MMV Nogueira

**Affiliations:** 1 Anesthesiology Department, Hospital das Clinicas, São Paulo, SP, Brazil; 2 Anesthesiology Department, Hospital do Servidor Público Estadual--IAMSPE, São Paulo, SP, Brazil; 3 Intensive Care Department, Hospital do Câncer de Barretos, São Paulo, SP, Brazil

## Introduction

Acidosis is a very frequent disorder in surgical patients. However, the nature of metabolic acidosis (hyperlactatemia, hyperchloremia, and others) seems to be indicative of worse clinical outcomes compared with pH value.

## Objective

This study assessed the role of different types of acidosis in the outcome of high-risk surgeries.

## Methods

Prospective, multicentric and observational study performed in three different tertiary hospitals. Patients who needed postoperative ICU admission were included in the study. Patients with low life expectancy (untreated cancer), hepatic failure, renal failure, and diabetes diagnosis were excluded. The patients were classified at ICU admission according to the presence and type of metabolic acidosis. The classification criteria were: base excess <-4 mmol/l; high albumin-corrected anion gap >12 mmol/l; and hyperlactatemia >2 mmol/l. Thus, acidosis was classified as hyperlactatemic, high or normal albumin-corrected anion gap (hyperchloremic).

## Results

**Figure 1:**
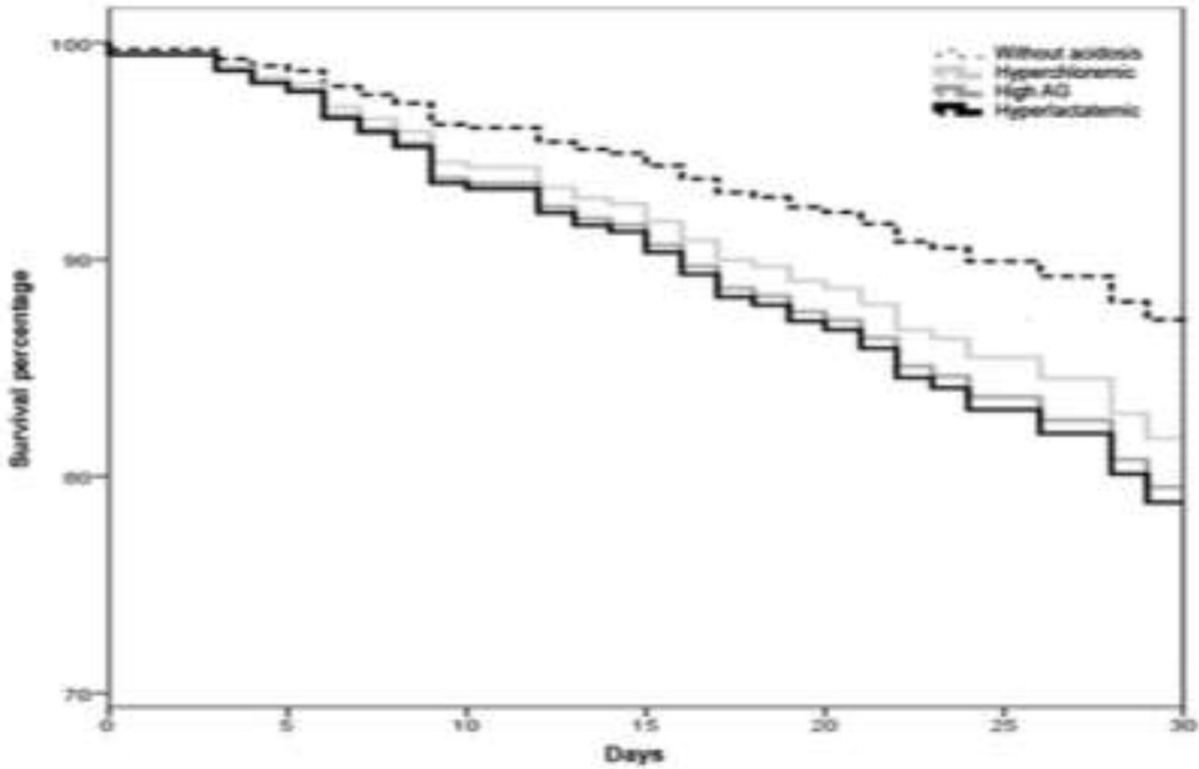
**Log rank test (Mantel-Cox, p = 0.03)**.

The study included 618 patients. Acidosis incidence at ICU admission was 59.1 %, being 148 (23.9 %) patients with hyperchloremia, 131 (21.2 %) with hyperlactatemia, 86 (13.9 %) with high anion gap, and 253 (40.9 %) without metabolic acidosis. Even though patients did not exhibit different demographic profile and severity, those who remained with acidosis after 12 hours, depending on group classification during the postoperative period, exhibited greater ICU complications: hyperlactatemia group = 68.8 %; high anion gap = 68.6 %; hyperchloremic = 65.8 %; and without acidosis = 59.3 %, *P *= 0.03. Cardiovascular, neurologic, and renal dysfunctions were the main complications and the hyperlactatemia group exhibited the highest level. The same result was observed with respect to hospital mortality rate, 30.1 % (HR 1.74, 95 % CI 1.02-2.96) hyperlactatemic; 24.3 % (HR 1.68, 95 % CI 0.85-3.81) high anion gap; 18.4 % (HR 1.47, 95 % CI 0.75-2.89) hyperchloremic; and 10.3 % no acidosis group (log-rank test--Mantel Cox, *P *= 0.03). See Figure 1.

## Conclusion

Metabolic acidosis in surgical patients is a highly prevalent postoperative complication, mainly related to hyperchloremia. Depending on the type, patients who developed metabolic acidosis postoperatively exhibited the worst outcomes compared with patients without acidosis, and the specific acidosis diagnosis can help in management.

